# HMGB1 targeting by ethyl pyruvate suppresses malignant phenotype of human mesothelioma

**DOI:** 10.18632/oncotarget.15152

**Published:** 2017-02-07

**Authors:** Laura Pellegrini, Jiaming Xue, David Larson, Sandra Pastorino, Sandro Jube, Kelly H. Forest, Zeyana Salim Saad-Jube, Andrea Napolitano, Ian Pagano, Vishal S. Negi, Marco E. Bianchi, Paul Morris, Harvey I. Pass, Giovanni Gaudino, Michele Carbone, Haining Yang

**Affiliations:** ^1^ University of Hawai'i Cancer Center, University of Hawai'i at Manoa, Honolulu, HI, USA; ^2^ University of Hawai'i at Manoa, Department of Cell and Molecular Biology, John A. Burns School of Medicine, Honolulu, HI, USA; ^3^ Leeward Community College, Mathematics and Sciences Division, University of Hawai'i System, Pearl City, HI, USA; ^4^ University of Hawai'i at Manoa, Myron B. Thompson School of Social Work, Office of Public Health and Center on Aging, Honolulu, HI, USA; ^5^ University of Hawai'i at Manoa, Department of Molecular Biosciences and Bioengineering, Honolulu, HI, USA; ^6^ San Raffaele University and Research Institute, Milan, Italy; ^7^ Department of Thoracic Surgery, Queen's Medical Center, Honolulu, HI, USA; ^8^ New York University School of Medicine, Department of Cardiothoracic Surgery, New York, NY, USA

**Keywords:** HMGB1, RAGE, ethyl pyruvate, mesothelioma, therapeutic

## Abstract

Human malignant mesothelioma (MM) is an aggressive cancer linked to asbestos and erionite exposure. We previously reported that High-Mobility Group Box-1 protein (HMGB1), a prototypic damage-associated molecular pattern, drives MM development and sustains MM progression. Moreover, we demonstrated that targeting HMGB1 inhibited MM cell growth and motility *in vitro*, reduced tumor growth *in vivo*, and prolonged survival of MM-bearing mice. Ethyl pyruvate (EP), the ethyl ester of pyruvic acid, has been shown to be an effective HMGB1 inhibitor in inflammation-related diseases and several cancers. Here, we studied the effect of EP on the malignant phenotype of MM cells in tissue culture and on tumor growth *in vivo* using an orthotopic MM xenograft model. We found that EP impairs HMGB1 secretion by MM cells leading to reduced RAGE expression and NF-κB activation. As a consequence, EP impaired cell motility, cell proliferation, and anchorage-independent growth of MM cells. Moreover, EP reduced HMGB1 serum levels in mice and inhibited the growth of MM xenografts.

Our results indicate that EP effectively hampers the malignant phenotype of MM, offering a novel potential therapeutic approach to patients afflicted with this dismal disease.

## INTRODUCTION

Human malignant mesothelioma (MM) is a rare, very aggressive cancer that arises from the transformation of the mesothelial cells lining the pleural, peritoneal and pericardial cavities [1]. MM causes about 43,000 deaths per year worldwide, and, in the United States, approximately 3,200 individuals are diagnosed annually with MM [1, 2]. MM development is primarily associated with occupational and environmental exposure to carcinogenic mineral fibers, such as asbestos and erionite [1]. SV40 has also been linked to MM as a co-carcinogen [3–5]. The chronic inflammation caused by asbestos deposition in tissues, leads to TNF-α release, which promotes tumor development [6, 7]. In addition, we recently discovered that BAP1 germline mutations play a critical role in MM pathogenesis [8, 9]. Somatic mutations in the BAP1 gene are also frequent in sporadic MM [10–12]. MM is a clinically challenging disease due to its resistance to most chemotherapies. Early detection is helpful and several biomarkers are being investigated [13, 14]. Despite multimodality therapy, the median overall survival is less than a year for patients with pleural MM, and a 5-year survival is observed in approximately 10% of patients diagnosed at early stages [1]. Therefore, the development and evaluation of new therapeutic approaches is highly needed.

HMGB1 is an abundant protein that has location-specific biological functions. In the nucleus, it acts as an architectural chromatin-binding factor, which stabilizes nucleosome formation and promotes protein assembly on specific DNA targets by bending the DNA [15, 16]. When localized to the cytoplasm, HMGB1 acts as a positive regulator of autophagy [17]. HMGB1 can be passively released by necrotic cells [18] and also actively secreted by inflammatory cells [19] and by some cancer cells [19–21] into the extracellular milieu, where it acts as a damage-associated molecular pattern (DAMP) and mediates inflammation or plays a role as chemo-attractant factor. Extracellular HMGB1 binds to multiple receptors (e.g. RAGE and TLRs) on inflammatory cells and leads to an increased secretion of pro-inflammatory cytokines, with the subsequent activation of pro-inflammatory and pro-tumoral transcription factors, such as NF-κB [reviewed in [22]].

We previously showed that HMGB1 has a key role in mesothelioma pathogenesis, as IT drives MM development and sustains MM progression [21, 23]. HMGB1 levels are significantly higher in the tissues and in the blood of MM patients, compared to healthy individuals [13]. Moreover, we found that HMGB1's receptor, RAGE, is unregulated in MM tumor cells, and most MM cells actively secrete high amounts of HMGB1 into the extracellular milieu and become “addicted” to it as they require HMGB1 for growth and survival [21]. HMGB1 is also upregulated and secreted in other cancer types, including prostate, colon, pancreas and breast cancers, and HMGB1 overexpression, in conjunction with its receptor RAGE, has been associated with a metastatic phenotype and linked to poor prognosis [24, 25]. Due to its critical role in cancer, in the last few years, several efforts have been made to identify both natural and synthetic compounds able to inhibit HMGB1 activities. Among them, the potential therapeutic use of ethyl pyruvate (EP), a potent HMGB1 inhibitor, has been proposed [26].

EP is a stable and lipophilic ester derived from the endogenous metabolite pyruvic acid [27]. EP has several pharmacological effects, such as the amelioration of redox-mediated cellular and tissue damage, the regulation of the apoptotic process, and the inhibition of inflammation [28–30]. EP also exerts protective effects against various inflammatory tissue injuries, such as lethal sepsis and systemic inflammation [26], hemorrhagic shock [29], and stroke [31]. Its effects are mainly associated with inhibition of NF-*κ*B activation and a reduction in the expression and secretion of pro-inflammatory cytokines. Due to this observation, subsequent studies have investigated EP's ability to inhibit the inflammatory mediator HMGB1 [26, 32, 33].

Besides its inflammation-modulatory functions, EP has been reported to affect tumor growth both *in vitro* and *in vivo*. In a liver and two melanoma tumor models, EP treatment significantly inhibited tumor growth [34, 35]. EP was also shown to facilitate a necrosis-to-apoptosis switch in glucose-deprived A549 lung adenocarcinoma cells via inhibiting HMGB1 release [33]. Moreover, EP-mediated down-regulation of the HMGB1-RAGE pathway was found to suppress invasive growth of gallbladder cancer cells [32].

Given the important role of HMGB1 in MM initiation and progression, and the current lack of effective therapeutic options for MM patients, we tested the hypothesis that EP may impair mesothelial cell transformation and suppress the malignant phenotype of MM.

## RESULTS

### EP affects the localization and secretion of HMGB1 in MM cells

We have previously demonstrated that HMGB1 is required for sustaining MM growth [21]. MM cells secrete high levels of HMGB1, which triggers an autocrine loop through its receptors-mainly RAGE-leading to cell proliferation and maintenance of the malignant phenotype. EP has been found to inhibit HMGB1 release [33, 34]. Therefore, we tested whether EP may modulate HMGB1 secretion in two MM cell lines, REN and HP3.

**Figure 1:**
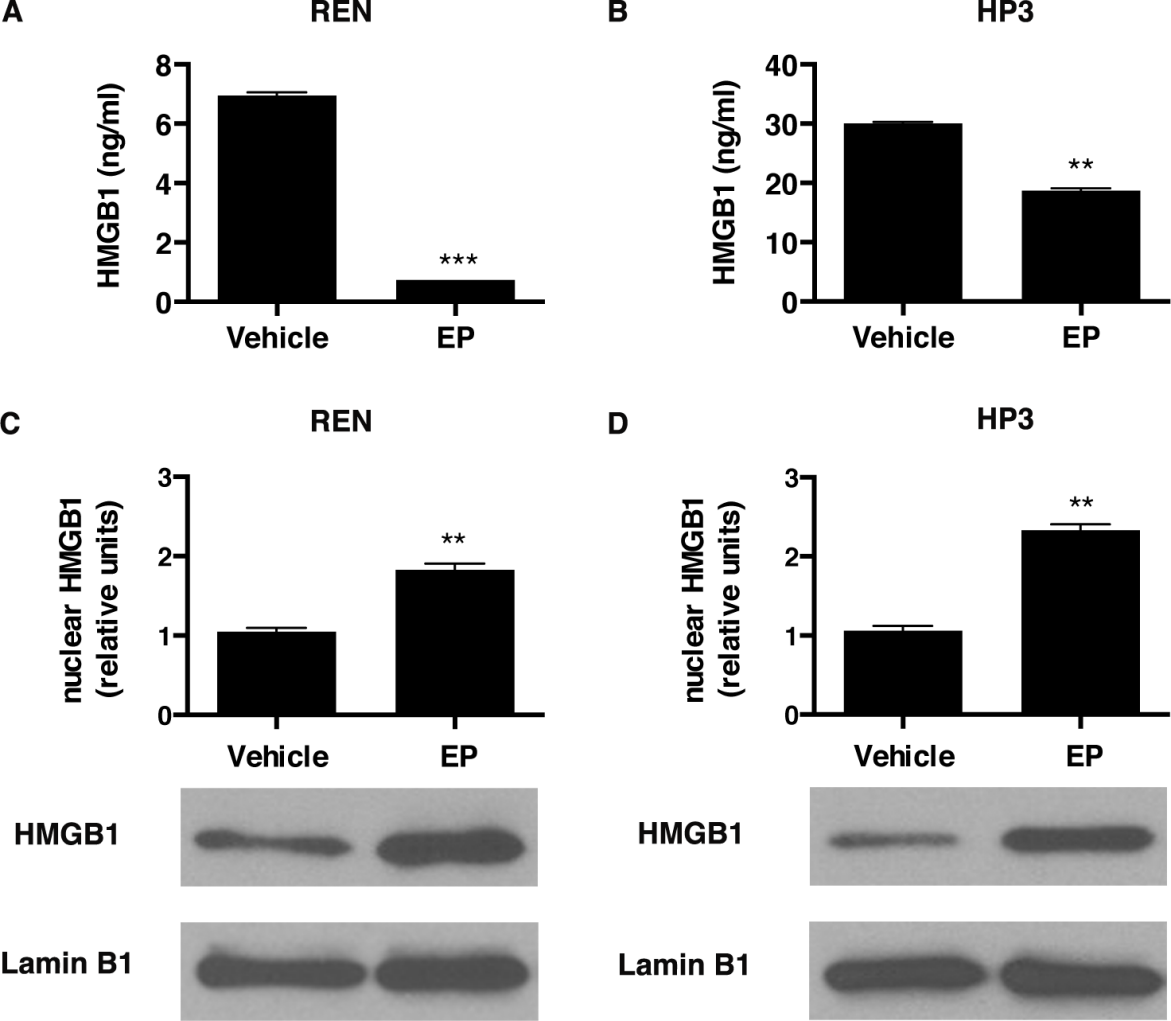
EP influences HMGB1 secretion and cellular localization REN (**A**) and HP3 (**B**) cells were treated with 5 mM EP for 48 h. Conditioned media were collected and HMGB1 levels were analyzed by ELISA. Experiments were performed in duplicate and carried out twice. Error bars represent SEM. **p < 0.01; *** *p* < 0.001. REN (**C**) and HP3 (**D**) cells were treated with 5 mM EP for 24 h. After the treatment cells were harvested and processed to extract the nuclear fractions. Lamin B1 was used as loading control. Histograms represent average HMGB1 levels relative to Lamin B1. Experiments were performed three times.

Cells were treated with EP, and after 48 hours a significantly lower amount of HMGB1 was detected in the cell culture medium, compared to vehicle-treated control cells (Figure 1A, 1B). Consistently, we observed an accumulation of HMGB1 in the nuclear fraction, in both MM cell cultures treated with EP (Figure 1C, 1D). These results suggested that EP was effective in inhibiting HMGB1 secretion in MM cells.

### EP impairs RAGE expression and NF-κB activity in MM cells

**Figure 2:**
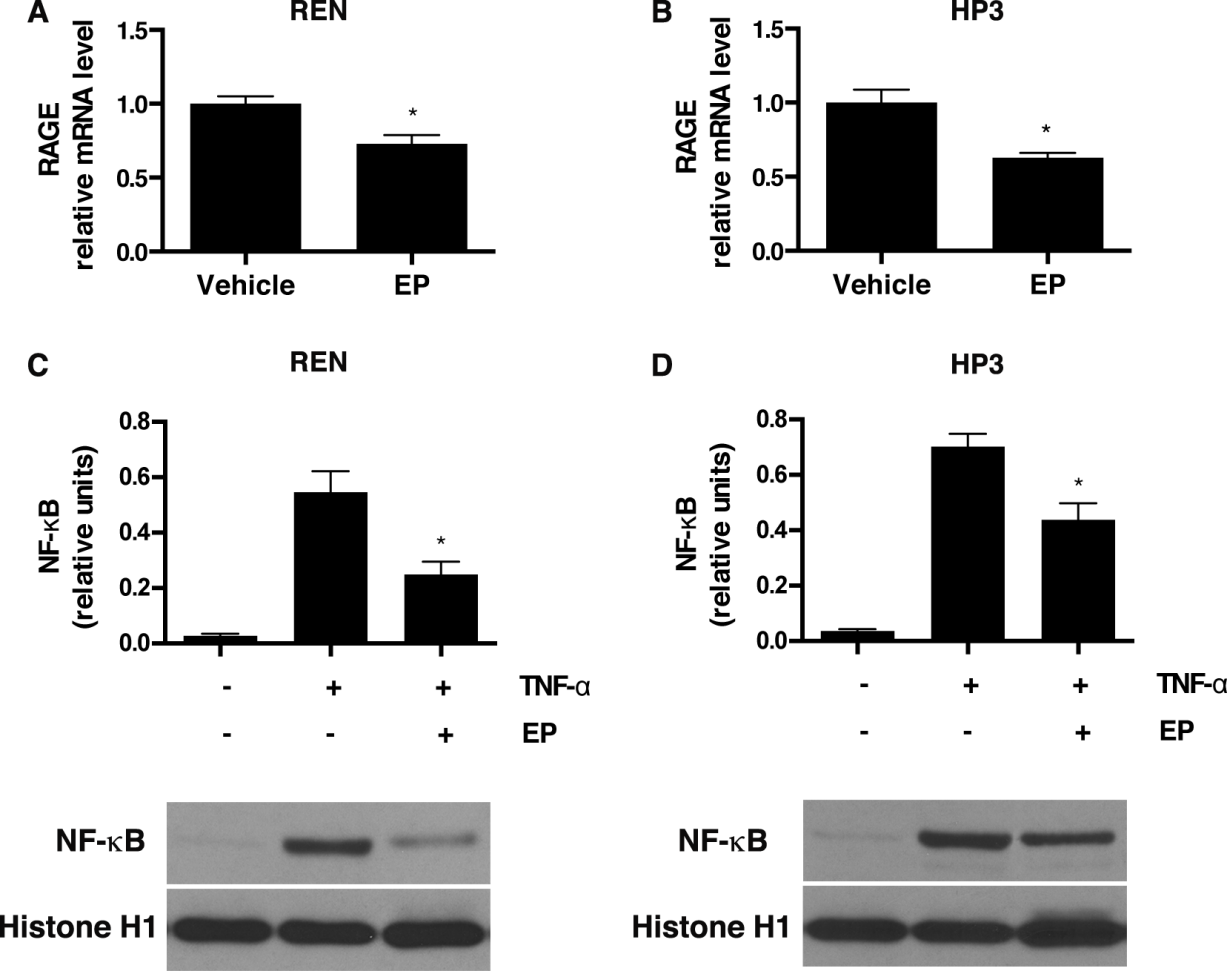
EP inhibits RAGE expression and NF-κB nuclear translocation (**A**) REN and (**B**) HP3 cells were treated with 5 mM EP for 48 h, and mRNA levels of RAGE were measured by RT-qPCR. *p < 0.05 (**C**) REN and (**D**) HP3 cells were pretreated with EP (2.5 mM) for 12 hrs, then stimulated with TNF-α (1 ng/ml) for 30 minutes. Cells were, then, harvested and the nuclear protein extracted and probed with NF-kB (p65) antibody. Histone 1 was used as a loading control. The intensity of NF-kB p65 bands is expressed as relative densitometry units. Experiments were performed in triplicate and repeated three times. Error bars represent SEM. *p < 0.05; TNF-α+EP versus TNF-α.

Activation of the HMGB1 signaling pathway leads to downstream upregulation of RAGE expression [36], which establishes an autocrine loop of activation that, in turn, sustains HMGB1 secretion and supports the survival of HMGB1-dependent cancers [21]. To test whether the effect of EP, on HMGB1 release, influences the HMGB1-RAGE signaling axis in MM, we evaluated the expression of RAGE in EP-treated REN and HP3 cells by RT-qPCR. The results indicated that treatment with EP for 48 h led to a significant decrease in RAGE mRNA levels in both cell lines (Figure 2A, 2B). The corresponding reduction in protein levels was further confirmed via Western Blot (Supplementary Figure 1A). To confirm the direct effect of EP in reducing HMGB1-induced expression of RAGE, REN cells were pretreated with EP for 3 h, followed by 24 h of stimulation with recombinant HMGB1, and RAGE mRNA expression was measured. As previously reported [37], we observed an increase in RAGE expression in cells treated with HMGB1, while in cells pretreated with EP, HMGB1-induced RAGE mRNA levels were significantly lower (Supplementary Figure 1B).

The HMGB1-RAGE signaling axis involves activation of NF-κB [38]. EP has been previously suggested to prevent HMGB1 release via NF-κB inhibition [26, 38]. Therefore, we investigated NF-κB p65 subunit translocation in MM, upon EP treatment. In both REN and HP3, the treatment with EP substantially inhibited TNF-alpha-mediated nuclear translocation of the NF-κB p65 subunit. This clearly indicates that EP inhibits NF-κB activation (Figure 2C, 2D) and suggests that NF-κB regulation is involved in the mechanism of EP-mediated inhibition of HMGB1 release and signaling.

Since our results suggested that EP effectively inhibited HMGB1 release and repressed the HMGB1-RAGE signaling axis in MM, this prompted us to test whether EP may affect MM tumorigenesis via targeting HMGB1.

### EP decreases viability, motility and migration of MM cells

**Figure 3:**
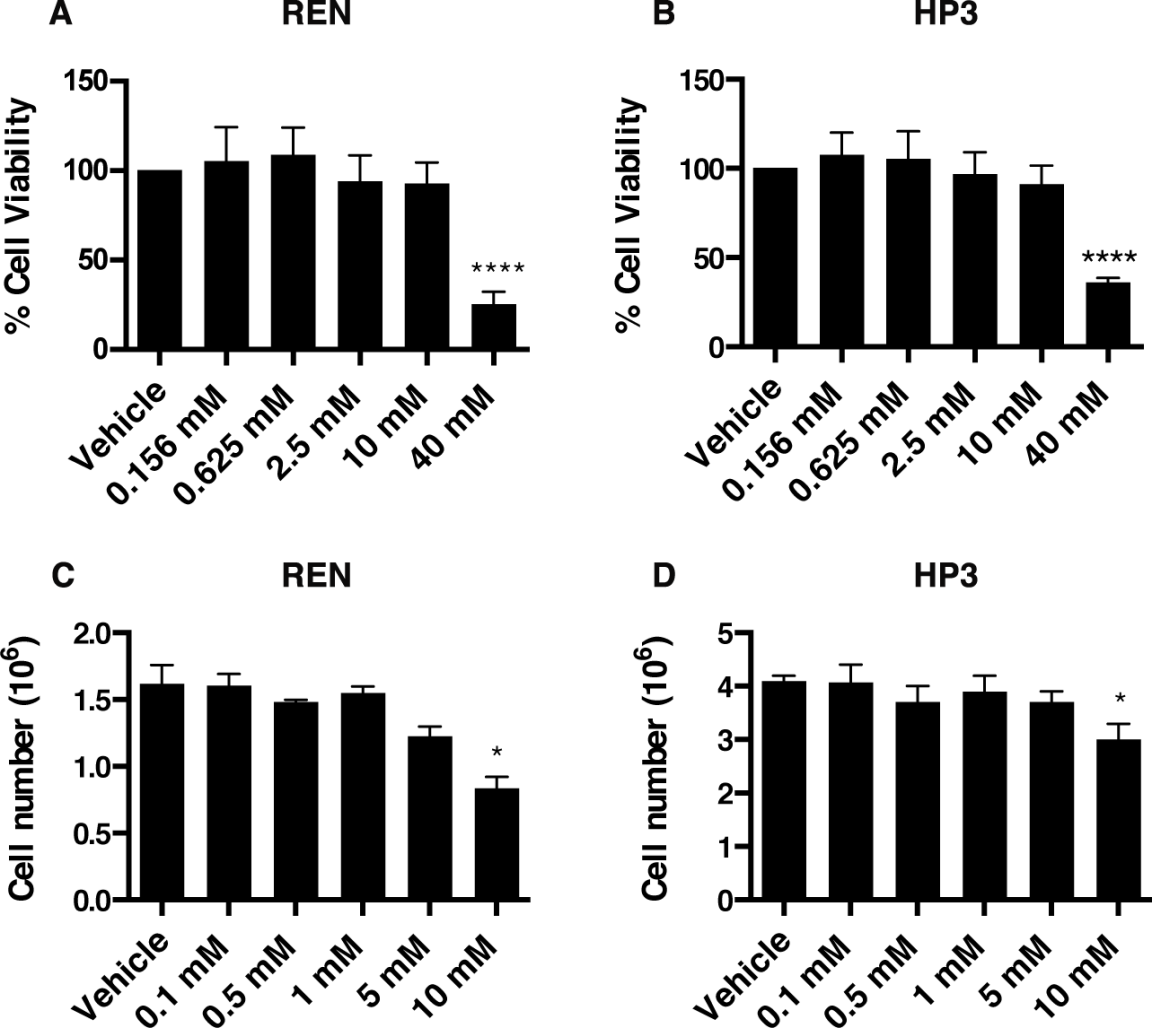
EP affects viability and cell number of MM cell lines Cell viability of REN (**A**) and HP3 (**B**) cells was determined by CyQUANT^®^ Cell Proliferation Assays. The assay was done in quadruplicate and performed twice. Manual cell counting of REN (**C**) and HP3 (**D**) cells after 5 days of treatment (EP different concentration). PBS was used as vehicle control. Error bars represent SEM. *p < 0.05; **** *p* < 0.0001.

To test whether EP influences MM tumorigenesis, we evaluated the viability and motility of REN and HP3 MM cells upon EP treatment. By using the CyQUANT^®^ Cell Proliferation Assay, we measured the survival rate of REN and HP3 cells exposed to increasing concentrations of EP for 24 h and 5 days. A significant reduction of viability was observed in both cell types, upon 24 h treatment, only using high doses of EP (40 mM) (Figure 3A, 3B), while 10 mM EP led to a decreased cell count only after 5 days of treatment (Figure 3C, 3D).

**Figure 4:**
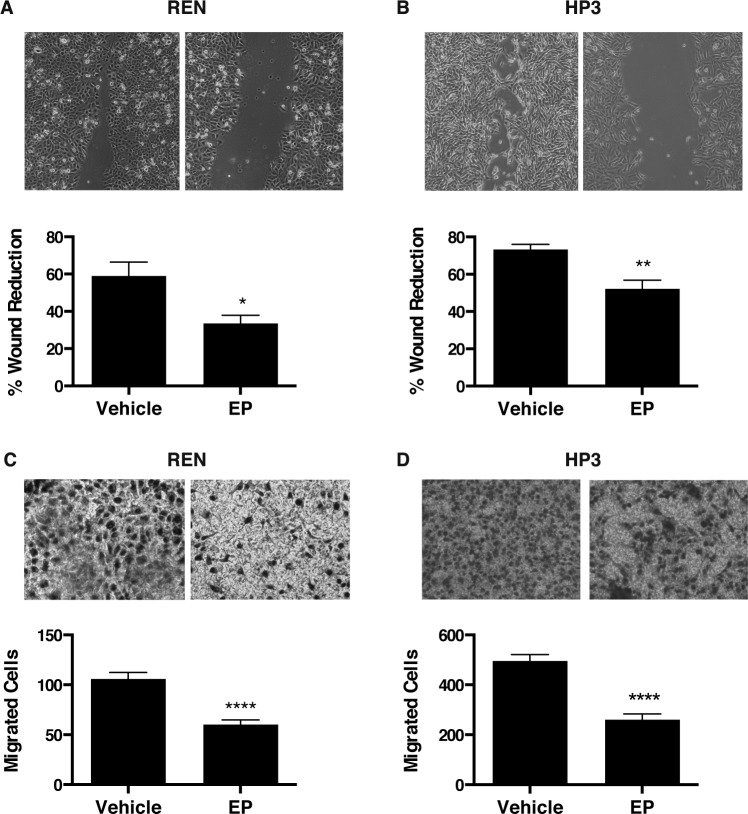
EP inhibits the motility and migration of MM cells Wound healing experiments were performed on confluent monolayer of (**A**) REN and (**B**) HP3 cells. The wounds were monitored and photographed after 48 h. Quantification of the percentage of wound reduction was analyzed using ImageJ software. Experiments were done in duplicate and performed twice. (**C**) REN and (**D**) HP3 cells migrated were stained and photographed after 48 h of treatment with vehicle or 10 mM of EP. The pictures were analyzed with Image J software. All experiments were done in duplicate and performed twice. Error bars represent SEM. *p < 0.05 **p < 0.01; **** *p* < 0.0001.

It has been previously shown that HMGB1 drives MM cell motility [21]. Therefore we performed wound-healing and migration assays to test whether EP influences MM motility in HMGB1-secreting MM cells, REN and HP3. We found that treatment with EP for 48 h significantly delayed wound closure and migration of REN and HP3 cells, as compared to vehicle (PBS)-treated controls (Figure 4A–4D). On the contrary, migration of PPM-MILL, a non-HMGB1 addicted MM cell line [21], was unaffected by EP treatment (Supplementary Figure 2). Together, these data indicate that EP effectively inhibits survival and motility of HMGB1-dependent MM cells *in vitro*. Differences were observed in sensitivity to EP for the different biological responses. Survival was inhibited only at high EP concentrations, while motility was affected by doses in the same range of inhibition of molecular parameters (protein, RNA).

### EP impairs anchorage-independent growth of MM cells

**Figure 5:**
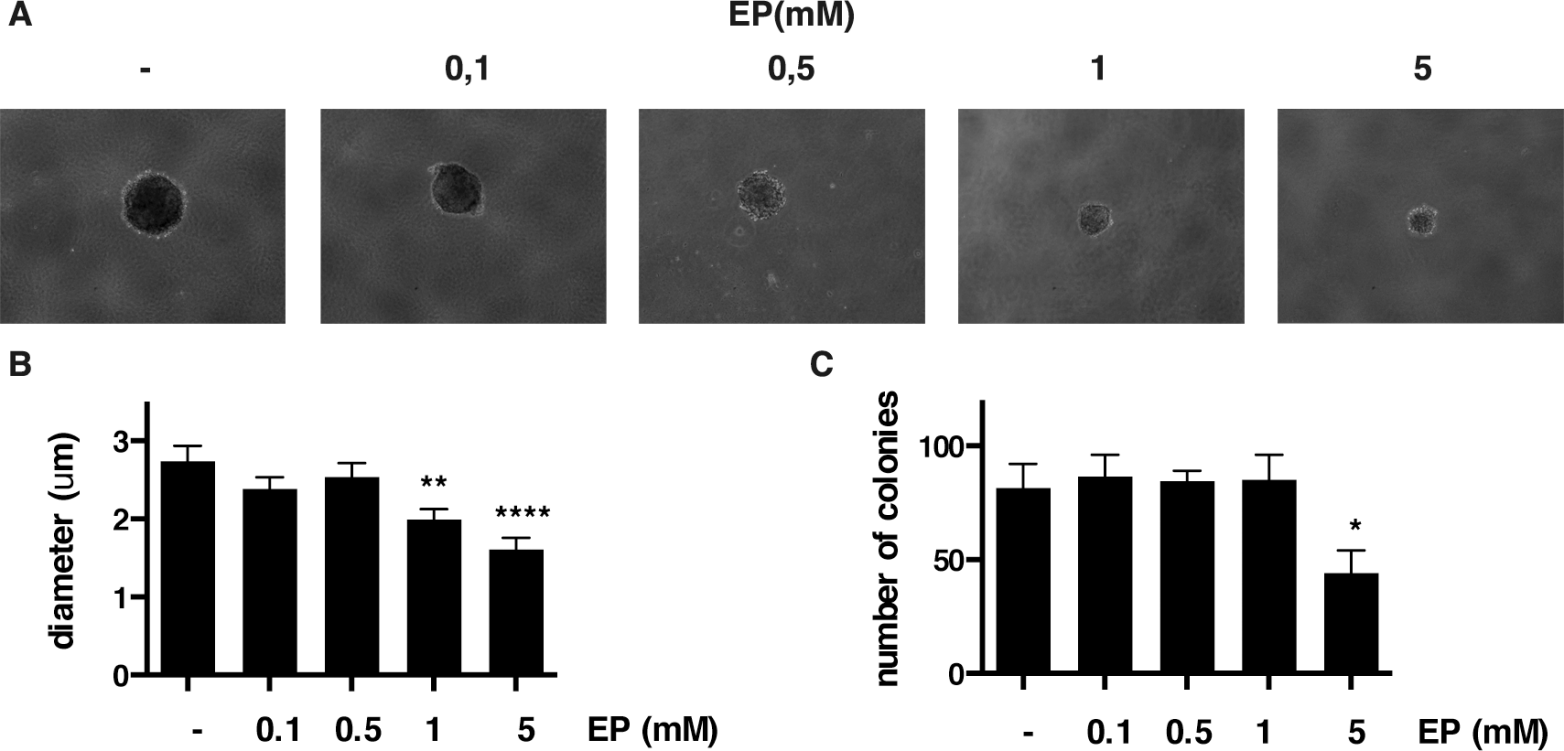
EP reduces colony formation of HP3 in soft agar Representative figure of HP3 colonies treated with vehicle or EP (**A**). For each condition the diameter (**B**) and the number of colonies (**C**) were determined using ImageJ software. The experiment was done in duplicate and repeated three times. Error bars represent SEM. *p < 0.05 **p < 0.01; **** *p* < 0.0001.

Next, we tested whether EP influences anchorage-independent growth of MM cells by soft agar assay. Compared to vehicle (PBS), EP caused a significant reduction of anchorage-independent growth in both REN and HP3 MM cells, as indicated by a marked reduction in number and size of the colonies (Figure 5A–5C and Supplementary Figure 3A, 3B). Of note, the effect was more pronounced in HP3 cells, in which a low concentration of EP (1 mM) was sufficient to significantly reduce anchorage-dependent growth.

These results revealed that EP interferes with a key aspect of the malignant phenotype of human MM cells, the anchorage-independent cell growth.

### EP decreases serum HMGB1 levels in MM xenografts, and inhibits orthotopic tumor growth

**Figure 6:**
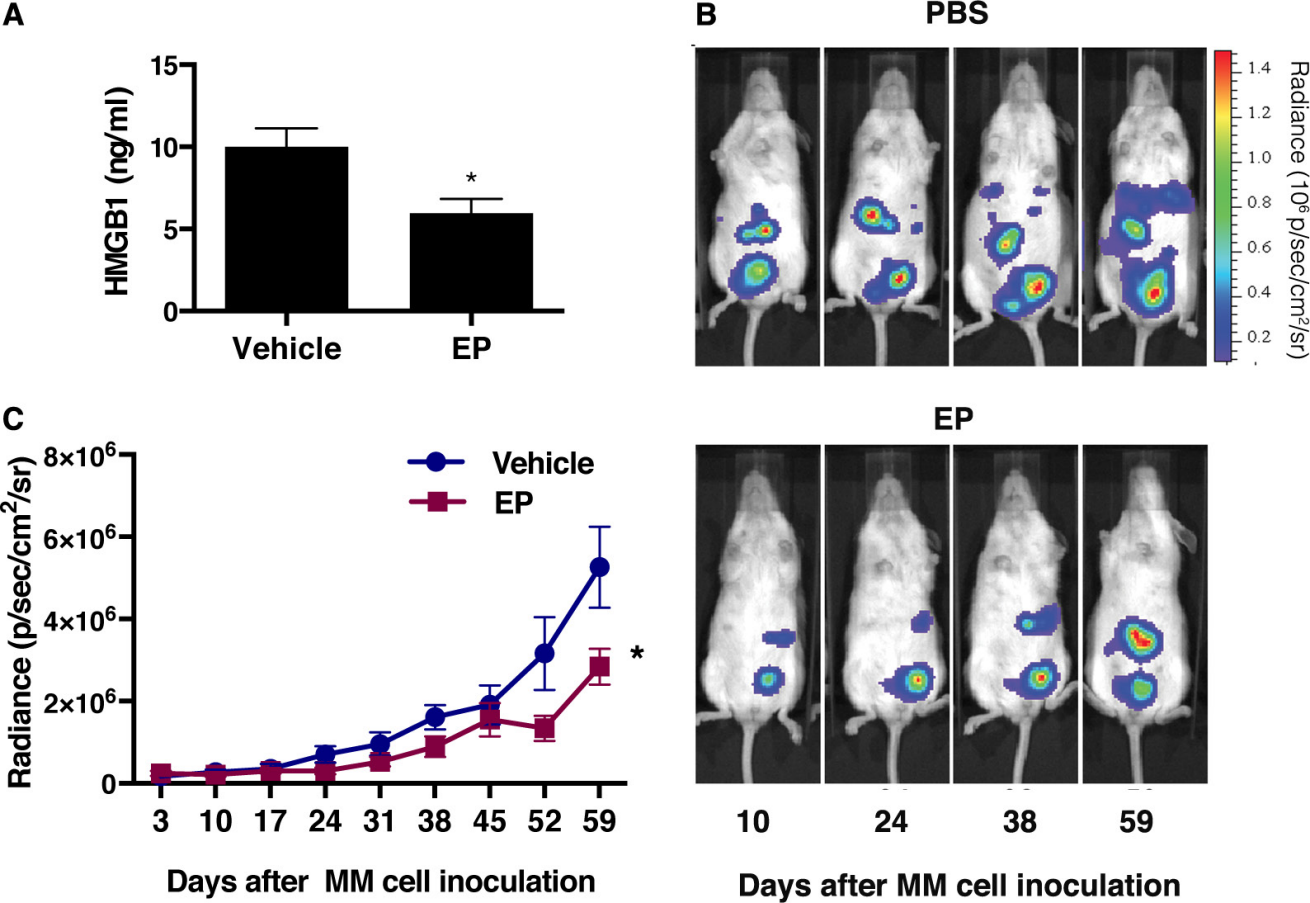
EP decreases serum levels of HMGB1 with concurrent reduction of the tumor growth (**A**) HMGB1 levels in severe combined immunodeficient (NOD.CB17-SCID) mice two months after the injection of REN/luc cells. (**B**) Xenografts were visualized by luminescence after D-luciferin injection (150 mg/kg) using the *In Vivo* Imaging System (IVIS^™^, Xenogen Corp., CA, USA), with regions of interest (ROI) quantified as total photon counts by Living Image software (Xenogen Corp.). One representative mouse from each group is shown. (**C**) Tumor size was measured and determined every 7th day, as average radiance (photons/s/cm2/sr). Data are represented as mean radiance values ± S.E.M. *p < 0.0001.

Based on the results indicating EP targeting HMGB1 *in vitro*, we evaluated whether EP may decrease HMGB1 and MM tumor growth *in vivo*. We powered our *in vivo* experiment to observe a difference in tumor size two months after initial injection, rather than a difference in overall survival because the latter would have required significantly more animals. Twenty mice were injected intra peritoneum (i.p.) with 5 × 10^5^ luciferase-expressing REN cells (REN/luc). Four days after MM cells injection, when tumor masses could be detected by *in vivo* bioluminescence imaging (IVIS), the mice were randomly assigned to EP treatment or control group. The treatment group received 2 mg EP/injection, three times a week for 8 weeks, while control group received 200 μl of PBS, with the same schedule as the treatment group. No systemic toxicity (body weight change) was observed upon EP treatment (Supplementary Figure 4A). Tumor growth was monitored by IVIS. Two months after the initial injection of MM cells, blood was drawn from each mouse and serum was isolated. HMGB1 serum concentration was evaluated by ELISA, and lower HMGB1 levels were found in the EP-treated group compared to untreated animals (Figure 6A). Consistently, the tumor volume was significantly smaller in the treated group [t(145) = 5.3, *p* < 0.0001] (Figure 6B, 6C). As expected, because of the small size of the animal groups, we were not able to detect a statistically significant difference in survival, even though a trend towards improved survival was observed in EP-treated mice (Supplementary Figure 4B).

These *in vivo* results further support HMGB1 as a pharmacological target for MM therapy. The results also suggest that EP may be a potential therapeutic agent for MM treatment.

### EP reduces foci formation *in vitro* and asbestos-induced release of HMGB1 *in vivo*

Based on our results in established MM tumor models, we speculated that EP might also have an inhibitory effect in asbestos-induced transformation of normal mesothelial cells due to the important roles of HMGB1 in asbestos carcinogenesis. We tested this hypothesis in vitro using our established human mesothelial (HM) and macrophages co-culture foci formation assay [39]. As shown in Supplementary Figure 5A, EP treatment significantly reduced the number of foci formed following asbestos exposure. Moreover, *in vivo*, we analyzed the level of HMGB1 in asbestos-treated mice and we found that EP significantly reduced HMGB1 serum levels (Supplementary Figure 5B). These results suggest that EP, by targeting HMGB1, can inhibit asbestos-mediated carcinogenesis and mesothelioma development.

## DISCUSSION

We demonstrated that EP impaired the malignant phenotype of MM cells *in vitro* and reduced the growth of MM *in vivo* using an orthotopic xenograft mouse model. Our data also indicated that the EP suppressive effect on MM was mediated by inhibition of the HMGB1-RAGE signaling axis.

EP is an anti-inflammatory agent and a HMGB1 inhibitor that has been shown to improve overall survival and to reduce organ dysfunction in a wide variety of inflammatory-related disease models. Due to the critical role of inflammation in both tumor initiation and progression, in recent years, several groups have studied the possible therapeutic effects of EP on cancer [32, 34, 35, 40–42]. It was found that EP is able to reduce tumor development and increase the overall survival of animals in different tumor models, such as liver, gastric and gallbladder. Some of the anti-tumor effects of EP were related to its ability to inhibit HMGB1 expression and/or secretion and to inhibit the HMGB1-RAGE signaling axis [32, 34, 40]. We have previously reported that MM cells express high levels of HMGB1 and its receptor RAGE, and that MM growth and progression requires these signaling molecules [21].

In the present study, upon EP treatment, we observed a significant reduction of HMGB1 secretion into the tissue culture medium, which was accompanied by an increase in the nuclear localization of HMGB1. Analysis of the localization of the p65 subunit of NF-κB suggested that the inhibition of HMGB1 release is mediated by the effect of EP on NF-κB activity.

Moreover, our data with RT-qPCR demonstrated that EP treatment specifically suppressed HMGB1-induced RAGE mRNA expression. Disruption of the HMGB1-RAGE autocrine loop of activation may reasonably explain the anti-tumor activity that we observed in MM using both *in vitro* and *in vivo* models. Using two different MM cell lines, we observed a reduction in MM growth, motility, migration, as well as an anchorage independent growth following EP treatment. EP significantly reduced MM xenograft growth, associated with decreased HMGB1 serum levels in EP-treated mice. Although our study was not able to evaluate how much of HMGB1 reduction *in vivo* was due to a direct EP inhibition of HMGB1 secretion and how much was due to an indirect effect because of lower tumor burden in EP-treated mice, our *in vitro* data support a model where direct inhibition of HMGB1 secretion by EP anticipates effects on tumor growth. Moreover, our results clearly suggest a beneficial effect of EP on MM despite the fact that our *in vivo* experiment was not powered to detect a significant difference in survival. Our work is the first report on the effects of EP on MM and indicates that EP may represent a reasonable therapeutic candidate for MM treatment.

The potential application of EP in cancer treatment is supported by its good safety and tolerability profile. Indeed, unlike other compounds, which inhibit or affect NF-κB pathway, EP has not been associated with host toxicity [43]. EP is commonly used as an additive in beverages and confectionary products, and its lack of toxicity has been demonstrated in extensive studies performed in animals [44]. Moreover, although a direct comparison of EP efficacy compared to other HMGB1 inhibitors is currently lacking, EP has been safely tested in humans and has been used in clinical trials [43]. In addition, some preclinical studies have demonstrated that inhibition of HMGB1 release by EP significantly increased the tumor cell sensitivity to other anticancer agents [17], and also protected against chemotherapy-induced cytotoxicity [45]. In summary, this evidence supports the possible use of EP as an adjuvant in MM treatment.

## MATERIALS AND METHODS

### Cell cultures

Malignant mesothelioma (MM) cell lines were established from surgically resected MM specimens. REN cells were provided by Dr. Steven Albelda (University of Pennsylvania, Philadelphia, PA) [46], whereas HP3 (also referred to as PHI) and PPM-MILL (originally referred to as H2373) were from our laboratories [47]. REN/luc luciferase was generated as previously described [48]. MM cells were routinely characterized and authenticated in our lab by immunostaining using antibodies against mesothelial markers (Wilms Tumor 1, calretinin and pancytokeratin) and in collaboration with Genetica DNA Laboratories (Cincinnati, OH, USA). Primary human mesothelial cells (HM) were obtained from pleural effusion of patients pathologically diagnosed free of malignancy, characterized and cultured as previously described [39, 49]. THP-1 human monocytes (ATCC, TIB202; ATCC, Manassas, VA) were cultured and differentiated into macrophages by phorbol 12-myristate 13-acetate (TPA), as previously described [21].

### Reagents and materials

EP, TPA and Human recombinant TNF-α were obtained from Sigma-Aldrich (St. Louis, MO, USA). The following primary antibodies were used: rabbit anti-HMGB1 (ab18256), rabbit anti-lamin B1 (ab16048), rabbit anti-RAGE (ab3611), mouse anti-GAPDH (ab8245), mouse anti-histone 1 (ab71594) (Abcam, Cambridge, MA, USA) and mouse anti-NF-kB p65 (sc-8008) (Santa Cruz Biotechnology, Santa Cruz, CA, USA). HMGB1 ELISA was obtained from IBL International (Hamburg, Germany).

Asbestos fibers were prepared as previously described [21, 50, 51]. Briefly, fibers were baked for 18 h at 150°C, added to 1× phosphate-buffered saline (PBS) solution, and passed through a 22-gauge needle 10 times to disaggregated fiber bundles.

### Western blotting

Cytoplasmic and nuclear fractions were separated from MM cells using a protein extraction kit (Active Motif, Carlsbad, CA), according to the manufacturer's instructions. Protein concentrations were determined using the Bradford method (Biorad, Hercules, CA, USA) and equal amount of protein lysate from each sample was separated on NuPAGE Novex 4–12% Bis-Tris mini gels (Invitrogen, Carlsbad, CA) and transferred to Hybond-C Extra nitrocellulose membranes (Amersham Biosciences, UK). The membranes were blocked in Tris-buffered saline containing 0.05% Tween 20 (TBST) and 5% skim milk at RT for 1 hours, then probed with the primary antibody at 4°C overnight. The membranes were, then, washed and incubated with the appropriate horseradish peroxidase-conjugated secondary antibody (Pierce, Rockford, IL) at RT for 1 hour. The signal was detected by enhanced chemiluminescence (Pierce). Experiments were performed three times.

### HMGB1 ELISA

The levels of HMGB1 in the sera of animals and in the conditioned media of MM cell lines were measured using the human HMGB1 ELISA kit (IBL International) following the manufacturer's protocol. The MM cell lines were cultured for 48 h in 1% DMEM plus PBS or 5 mM EP. The culture media were then collected and concentrated by ultrafiltration using Amicon Ultra Centrifugal Filters (Millipore). Protein concentration was determined using the Bradford method (Biorad, Hercules, CA, USA) and for each condition, equal amount of protein was analyzed.

### RNA extraction, RT-PCR and quantitative real-time PCR

Total RNA was isolated from the cultured cells with Trizol reagent (Invitrogen, Carlsbad, CA) and quantified using micro-spectrophotometry (Nano-Drop Technologies, Inc.). Total RNA was reverse transcribed (RT) using the high capacity cDNA reverse transcription kit (Invitrogen, Carlsbad, CA) according to manufacturer's protocols. RT-qPCR was performed with TaqMan probes and TaqMan universal PCR Master Mix (Invitrogen, Carlsbad, CA). The amplification was carried out with an ABI PRISM 7900 RT-PCR System. Triplicate assays were performed with RNA samples isolated from at least two independent experiments. Fold changes in gene expression were calculated using the ΔΔCt method using β-actin as the normalization control.

### Viability and cytotoxicity assays

MM cells (1 × 10^3^ per well) were seeded in a 96-well tissue culture plate and incubated for 24 hours in DMEM with 1% FBS containing EP at different concentrations. PBS was used as vehicle control. Cell viability was determined using the CyQUANT^®^ Cell Proliferation Assays (Life Technologies, NY, USA) following the manufacturer's instruction. The assay was done in quadruplicate and performed twice.

MM cells (1 × 10^4^ per well) were seeded in a 6 wells plate and incubated for 5 days in DMEM with 1% FBS containing EP at different concentrations. PBS was used as vehicle control. Cells were manually counted using a hemocytometer.

### Wound healing assay

MM cells were seeded in 6-well plates and grown to 80% to 90% confluence in DMEM plus 1% FBS. The cell monolayer was carefully wounded with a P200 pipette tip, then washed to remove cell debris and treated with EP (10 mM) or vehicle (PBS) control. The wounds were observed and photographed after 48 h. For quantification of wound closure, the scratched area covered by the cells was measured using ImageJ software and normalized to control. Experiments were done in duplicate and performed three times.

### Migration assay

The *in vitro* cell migration assay was carried out using Costar Transwell^®^ permeable polycarbonate supports (8.0-μm pores) in 24-well plates (Corning Inc., NY, USA). For this assay, 1 × 10^5^ MM cells in 200 μl serum-free DMEM were seeded in the upper compartment of the Transwell^®^ system. The lower compartment contained 10% FBS DMEM plus PBS or 10% FBS DMEM supplemented with EP (10 mM). The plate was incubated at 37°C, and the cells were allowed to migrate into the lower compartment for 48 h. Then the media and the cells on the upper surface of the membrane were removed with a cotton swab, and the cells on the lower surface were stained using HEMA 3 staining kit (Millipore, MA, USA). The migrated cells were visualized under light microscope and counted from three random fields using the ImageJ software. Experiments were done in duplicate and performed three times.

### Soft agar assay

Anchorage-independent cell growth was determined by the soft agar assay. MM cells (4 × 10^3^) were mixed with 0.6% agar in DMEM plus 10% FBS (1:1) and placed on top of a 6-well plate precoated with 1.2% agar in DMEM plus 10% FBS (1:1). Cells were cultured at 37°C with 5% CO_2_ and fresh medium (DMEM plus 1% FBS) supplemented with different concentration of EP was added every two days. After 25 days of culture, the number and size of the colonies formed in each treatment were evaluated. For each well, all colonies larger than 0.1 mm in diameter were counted using ImageJ software. Experiments were done in duplicate and performed twice.

### SCID orthotopic human MM xenografts

Twenty severe combined immunodeficient (NOD.CB17-SCID) female mice aged 6 to 8 weeks (Jackson Laboratories, Bar Harbor, ME) were housed and handled under aseptic conditions, in accordance with our institution's Institutional Animal Care and Use Committee (IACUC) guidelines. The animals were injected intra peritoneum (i.p.) with REN/luc cells (5 × 10^5^) suspended in 500 μl of PBS, as described [48, 52]. Xenografts were visualized by luminescence after D-luciferin injection (150 mg/kg) using the In Vivo Imaging System (IVIS^™^, Xenogen Corp., CA, USA), with regions of interest (ROI) quantified as total photon counts by Living Image software (Xenogen Corp.). Four days were required for the formation of detectable tumor nodules by IVIS imaging. Mice were then randomly assigned to control (PBS) and treatment (EP) groups of ten animals each. Animals in the EP group received 2 mg (100 mg/kg/day) EP/injection i.p. three times a week, for 8 weeks (for a total of 48 mg EP/mouse). The dosage of EP used *in vivo* was determined based on previously published studies [53]. The control group received i.p. injections of 200 μl vehicle (PBS) with the same schedule as the treatment groups. Tumor dimension was measured and determined every 7th day as the average radiance (photons/s/cm^2^/sr). Two months after the beginning of the experiment, blood was drawn from animals in all groups and the sera collected and used for the detection of HMGB1 levels by ELISA.

### *In vitro* co-culture asbestos-induced transformation assay

Primary HM cells (3 × 10^5^) were seeded in 6-well plates and co-cultured with phorbol 12-myristate 13-acetate (TPA)-differentiated macrophages. Differentiated macrophages were co-cultured in an insert chamber (BD Falcon, Bedford, MA) placed on the top of the HM. The bottom of the insert chamber has 0.4-μm pores that allow cytokines and growth factors produced by macrophages to reach HM cells cultured in the lower chamber.

Crocidolite (5 μg/cm^2^) was then added into the co-culture system. EP (0.5 mM) was added twice a week together with fresh media and freshly differentiated macrophage cells for a total of 8 weeks. After that time, foci were analyzed by crystal violet staining and were counted under a light microscope.

### *In vivo* asbestos exposure

BALB/cAnNCrl (BALB/c) female mice aged 3 to 4 weeks (Charles River Laboratories, Wilmington, MA) were housed and handled under aseptic conditions, in accordance with our institution's Institutional Animal Care and Use Committee (IACUC) guidelines. The animals were randomly assigned to negative control (vehicle/PBS), positive control (crocidolite) and treatment (crocidolite+EP) groups of 5 animals each. Mice in the treatment group were pre-treated for 3 days with 200 μl i.p. injections of EP (100 mg/kg/day), then the positive control and treatment group were injected i.p. with 1 mg crocidolite asbestos. After asbestos injection, animals in the treatment group were given 200 μl i.p. injections of EP (100 mg/kg/day) three times a week for 3 weeks, while animals in the control groups received 200 μl vehicle (PBS) with the same schedule as the EP-treated group. Blood was drawn from all groups at weeks 0, 1, 2 and 3 after asbestos injection; sera were then collected and used for the detection of HMGB1 levels by ELISA. Following completion of the treatment period, mice were euthanized according to IACUC regulations.

### Statistical analysis

Where not otherwise indicated, statistical significance between 2 groups of interest was evaluated by unpaired Student *t* test. Differences were considered significant at *P* < 0.05. To compare tumor growth rates between the EP and control groups, we ran mixed (repeated observations over time) polynomial regression analysis, which accounted for nonlinear growth. The model outcome was tumor radiance and the predictors were group (EP vs. control), days of growth, and the square of days. To assess group differences in growth rate, the interaction term between group and days was included, providing a slope comparison test. Tumor size was assumed to be zero at zero days, so no intercept was included. The Mixed procedure in the SAS 9.4 software (SAS Institute Inc., Cary, NC) performed the statistical analysis.

## SUPPLEMENTARY MATERIALS FIGURES AND TABLES



## Abbreviations

HMGB1: High-mobility group box-1
MM: malignant mesothelioma
EP: Ethyl pyruvate
RAGE: receptor for advantage glycation end products
DAMP: damage associated molecular pattern
TLR: Toll like receptor
HM: human mesothelial
TPA: 12-*O*-Tetradecanoylphorbol-13-acetate
