# Supplementary material for: HMGB1 targeting by ethyl pyruvate suppresses malignant phenotype of human mesothelioma
Source: Oncotarget. 2017 Feb 7;8(14):22649–61. doi: 10.18632/oncotarget.15152 (PMC5410252; doi:10.18632/oncotarget.15152)
Supplement: Supplementary file 1 [file oncotarget-08-22649-s001.pdf]

HMGB1 targeting by ethyl pyruvate suppresses malignant phenotype of human mesothelioma

Supplementary Materials

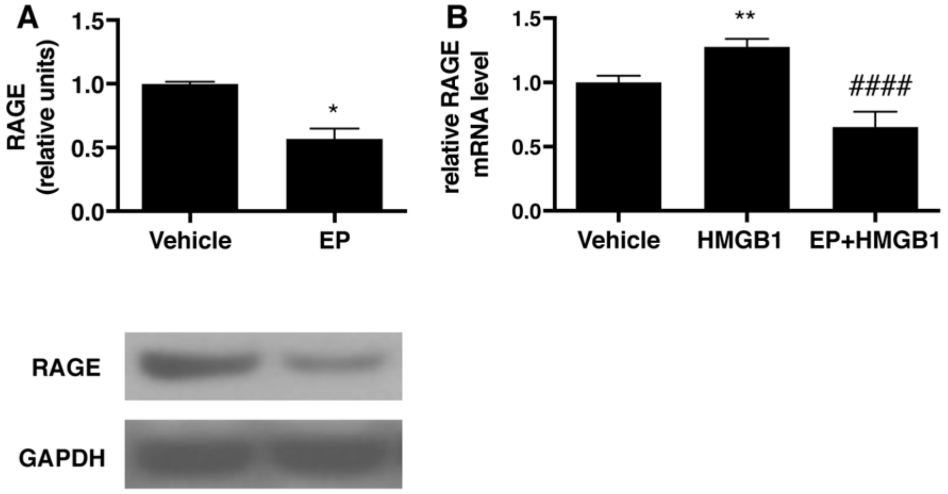

Supplementary Figure 1: EP reduces RAGE protein levels. (**A**) REN cells were treated with 5 mM for 48 h, and protein levels of RAGE were measured by Western Blot. GAPDH was used as a loading control. Histograms represent average RAGE levels relative to GAPDH. Data show a representative experiment out of three performed. (**B**) REN cells were pretreated with 10 mM EP for 3 h, and then stimulated with 100 ng/ml of recombinant HMGB1 for 24 h. After treatment the mRNA level of RAGE were measured by real time PCR. The assay was done in triplicate and β-actin was used as normalization control. Error bars represent SEM. $^{**}p < 0.05$ compared to REN cells treated with vehicle; $^{\#\#\#\#}p < 0.0005$ compared to REN cells treated with recombinant HMGB1.

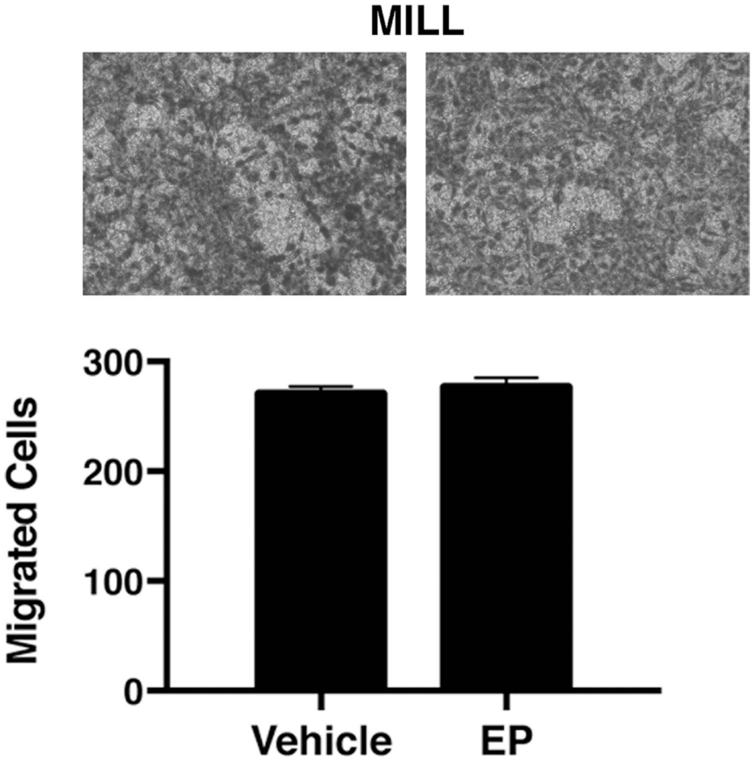

Supplementary Figure 2: EP does not affect the migration of a non HMGB1-addicted cell line. Migrated PPM-MILL cells were stained and photographed after 48 h of treatment with vehicle or 10 mM of EP (original magnification 100x). The pictures were analyzed with Image J software. The experiment was done in duplicate and performed twice.

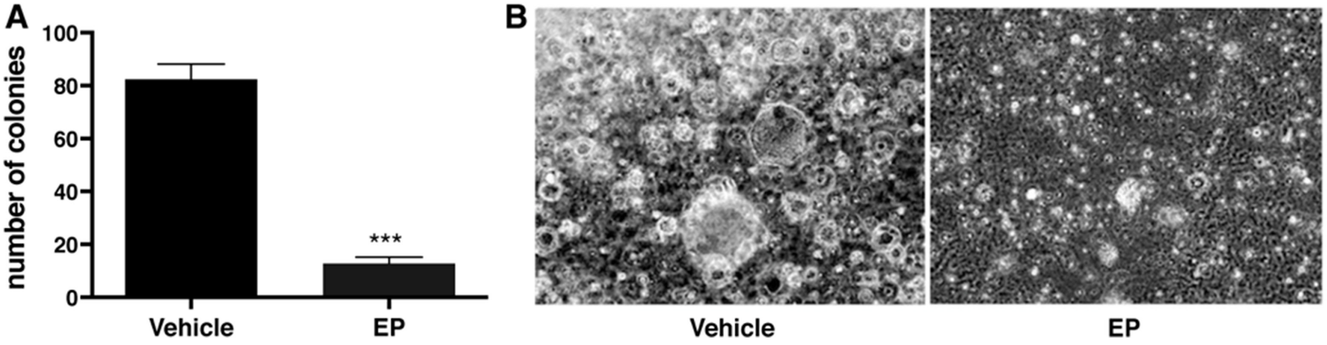

Supplementary Figure 3: EP reduces colony formation of REN cells in soft agar. REN cells were seeded on soft agar-coated plates. Fresh medium (DMEM plus 1% FBS) supplemented with 5 mM EP or vehicle control was added every 2 days for 23 days. (**A**) Quantification of number of colonies and (**B**) representative photographs of colonies. The number of colonies was calculated using ImageJ. The experiment was done in duplicate and repeated three times. Error bars represent SEM. ***$p < 0.001$.

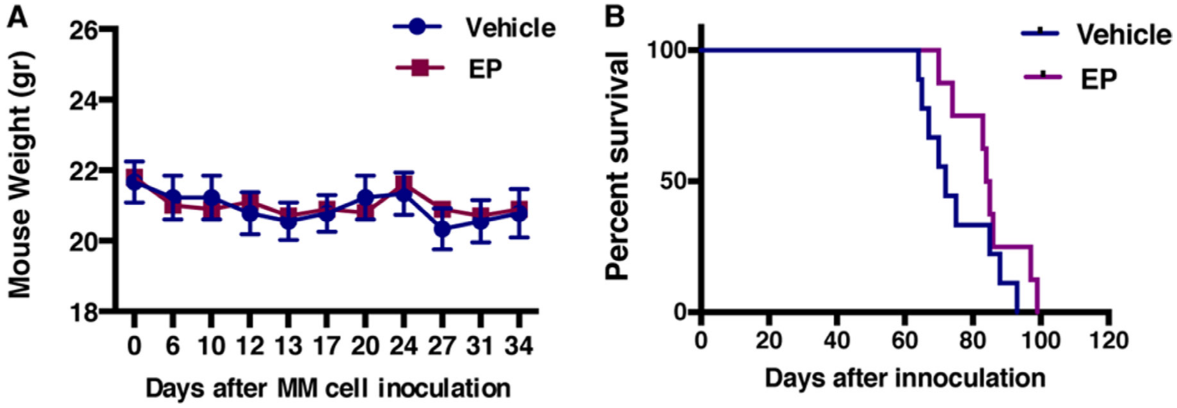

Supplementary Figure 4: Body weight change and survival curve. Severe combined immunodeficient (NOD.CB17-SCID) mice were injected with REN/luc cells. Mice in the treated group received 2 mg EP/injection, three times a week for 8 weeks, while control group received 200 µl of PBS, with the same schedule. (**A**) Change in body weight (expressed in grams), over time, in PBS and EP treated mice. (**B**) Survival curves of PBS or EP treated animals were compared using log-rank test ($p = 0.15$).

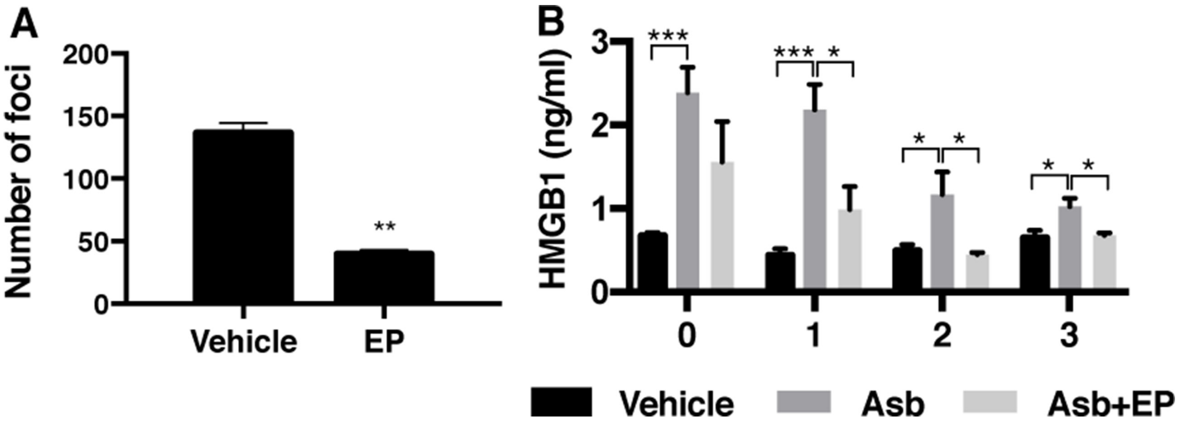

Supplementary Figure 5: EP hinders asbestos-induced HM transformation in vitro and decreases plasma HMGB1 concentration upon asbestos exposure *in vivo*. (**A**) Foci formation. HM cells were co-cultured with macrophages, exposed to crocidolite asbestos (5 µg/cm^2) and treated with EP (0.5 mM). After 2 months, the number of foci developed in the co-cultures was counted. Experiments were performed twice. Error bars represent SEM. *$P < 0.05$ EP versus vehicle (PBS). (**B**) Plasma HMGB1 concentration. BALB/c female mice were randomly assigned to negative control (vehicle/PBS), positive control (crocidolite) and treatment (crocidolite+EP) groups of 5 animals each. Mice in the positive control and treatment groups were injected i.p. with 1 mg crocidolite asbestos. After crocidolite injection, animals in the treatment group were given 200 µl i.p. injections of EP (100 mg/kg/day) every other day for 17 days for a total of 9 injections, while animals in the control groups received 200 µl vehicle (PBS) with the same schedule as the EP-treated group. Blood was drawn from the animals in all groups at weeks 0, 1, 2 and 3 after crocidolite injection; the sera was then collected and used for the detection of HMGB1 levels by ELISA. *$P < 0.05$; asbestos+EP versus asbestos.
